# Precise preoperative diagnosis of struma ovarii with pseudo-Meigs’ syndrome mimicking ovarian cancer with the combination of ^131^I scintigraphy and ^18^F–FDG PET: case report and review of the literature

**DOI:** 10.1186/s13048-018-0383-2

**Published:** 2018-02-02

**Authors:** Sayaka Fujiwara, Hideaki Tsuyoshi, Toshiya Nishimura, Nozomu Takahashi, Yoshio Yoshida

**Affiliations:** 1 Department of Obstetrics and Gynecology, Kizawa Memorial Hospital, 590 Shimokobi, Kobi-cho, Minokamo-shi, Gifu 505-8503 Japan; 2 Department of Obstetrics and Gynecology, Faculty of Medical Sciences, University of Fukui, 23-3 Shimoaizuki, Matsuoka, Eiheiji-cho, Yoshida-gun, Fukui 910-1193 Japan

**Keywords:** Struma ovarii, Pseudo-Meigs’ syndrome, ^18^F–FDG PET, ^131^I scintigraphy

## Abstract

**Background:**

Struma ovarii is a rare ovarian neoplasm that often appears malignant on conventional imaging. Pseudo-Meigs’ syndrome with ascites, pleural effusion, and elevated serum CA 125 levels is much rarer and leads to misdiagnosis of ovarian cancer and unnecessary extended surgery.

**Case presentation:**

A 50-year-old woman with abdominal distention and dyspnoea was referred to our hospital. Ultrasound, computed tomography (CT), and magnetic resonance imaging (MRI) showed a polycystic ovarian tumor with a solid component, pleural effusion, and massive ascites with negative cytology. Her serum CA 125 level was 1237 U/ml, indicating the presence of ovarian cancer. Based on increased uptake of ^131^I but no uptake of ^18^F–FDG in the tumor, the preoperative diagnosis was struma ovarii with pseudo-Meigs’ syndrome, which was confirmed histologically. She had no evidence of ascites and pleural effusion six months after surgery.

**Conclusions:**

To date, there have been no systematic reviews focused on preoperative diagnosis with imaging modalities. The combination of ^131^I scintigraphy and ^18^F–FDG PET/CT in addition to conventional imaging modalities can provide the precise preoperative diagnosis of struma ovarii with pseudo-Meigs’ syndrome mimicking ovarian cancer, leading to the appropriate treatment strategy.

## Background

Struma ovarii is a rare ovarian neoplasm categorized as a variety of mature cystic teratoma and composed predominantly of thyroid tissue. This tumor often has the appearance of ovarian cancer with a solid component or thick septa in a polycystic tumor, although it is generally benign. Moreover, it can sometimes be associated with massive ascites and pleural effusion, called pseudo-Meigs’ syndrome, leading to the preoperative misdiagnosis of malignancy. To date, preoperative diagnosis with conventional modalities including ultrasound (US), computed tomography (CT), or magnetic resonance imaging (MRI) has been attempted. However, most cases reviewed were misdiagnosed as advanced ovarian cancer, and some of them underwent unnecessary extended surgery. Thus, an optimal diagnostic strategy is needed. Herein, the successful preoperative diagnosis of struma ovarii with pseudo-Meigs’ syndrome with the combination of ^18^F–FDG PET/CT and ^131^I scintigraphy is reported.

## Case presentation

A 50-year-old woman (gravida 1, para 1) visited the hospital due to abdominal distension, anorexia, and exertional dyspnoea. She had been in good health and postmenopausal for 1 year. Physical examination showed a markedly distended abdomen. Abdominal US showed a pelvic mass and gross ascites. She was referred to our hospital for further examination and subsequent surgery.

**Fig. 1:**
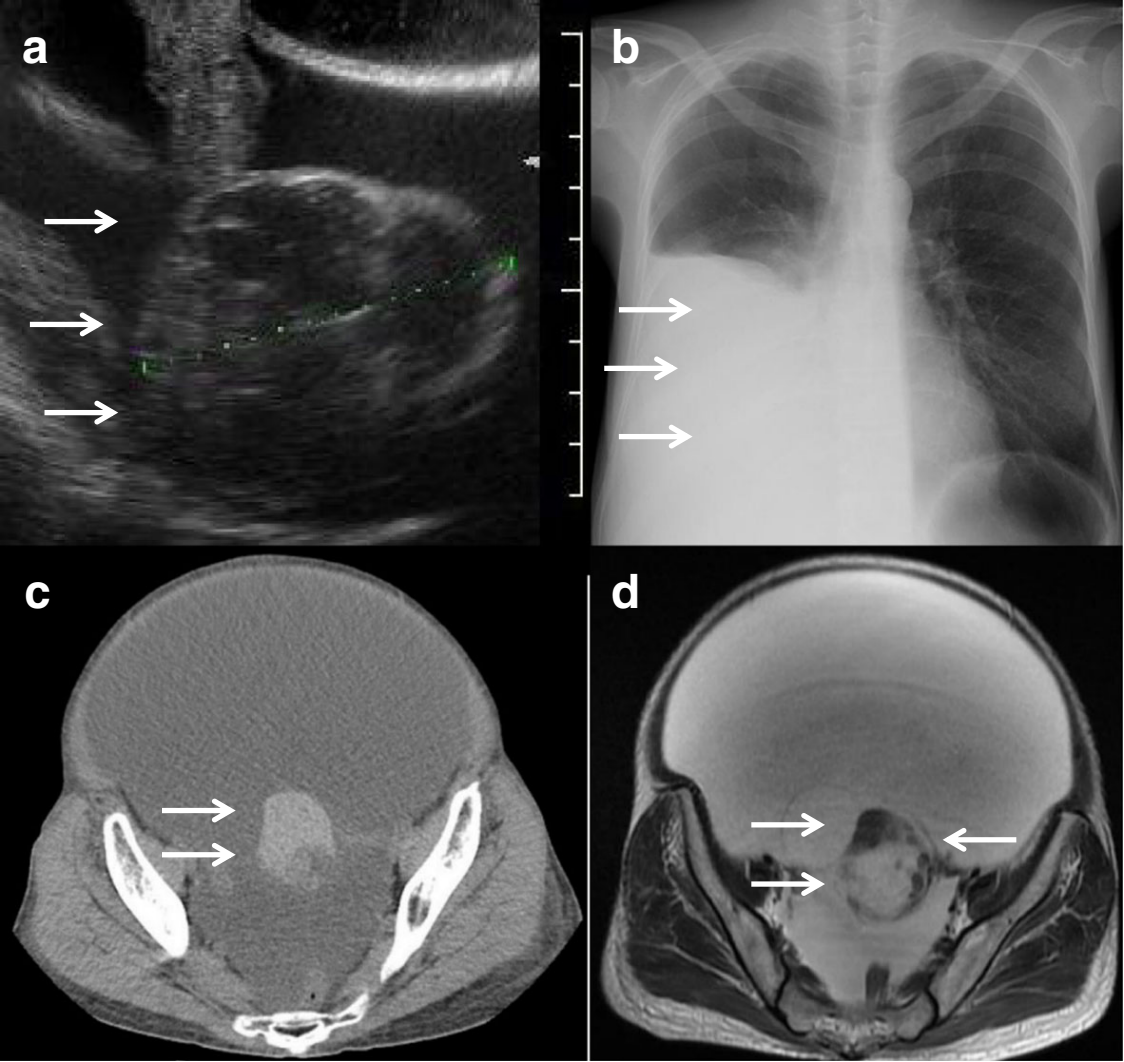
Transvaginal US showing marked ascites and a large solid and cystic mass with a diameter of 8 cm in the left ovary (arrows) (**a**). Chest X-ray showing a massive right-sided pleural effusion (arrows) (**b**). CT scans showing a solid component of the tumor with a higher attenuation lesion (arrows) (**c**). T2-weighted MRI showing the cystic lesions with various intensities separated by the septa (arrows) (**d**)

Transvaginal US showed the presence of marked ascites and a large solid and cystic mass with a diameter of 8 cm in the left ovary (Fig. 1a). Chest X-ray confirmed a right-sided pleural effusion without lung metastases (Fig. 1b). CT of the abdomen and pelvis showed gross ascites that extended under the diaphragm and a heterogeneous mass in the left adnexa. On precontrast scans, a part of the solid component had higher attenuation than the other part (Fig. 1c) and was strongly enhanced. It also had a small part with calcifications along the cystic wall. There was no lymphadenopathy or peritoneal dissemination. On pelvic MRI, the uterus and right adnexa were unremarkable. The left pelvic mass showed homogeneous low intensity on T1-weighted MRI and heterogeneous low and high intensities on T2-weighted MRI, suggesting the presence of cystic and solid lesions. The cystic lesions had various intensities on T2-weighted MRI and were separated by thickened septa (Fig. 1d). Solid parts including the thick septa were uniformly low intensity on T2-weighted MRI and well enhanced on contrast-enhanced MRI. There were no fatty components. The patient’s serum CA 125 level was 1237 U/ml (normal value < 35 U/ml). Serum CEA, CA 19–9, and SCC levels were within normal ranges. The pelvic cyst with solid components, the high CA 125 level, massive ascites, and pleural effusion strongly indicated the presence of an advanced ovarian cancer.

**Fig. 2:**
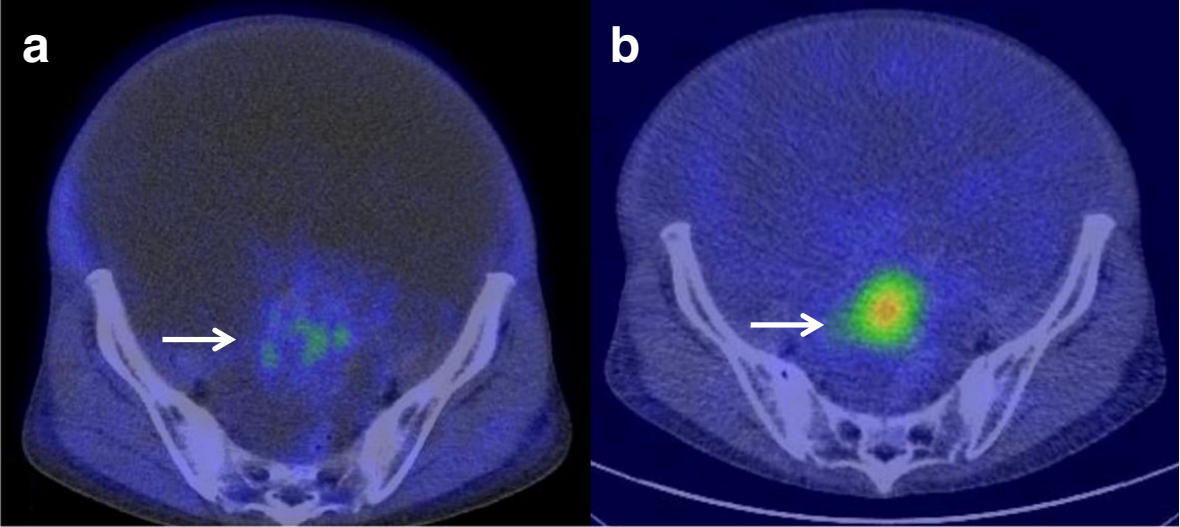
Pelvic FDG PET/CT shows lesions in the tumor without strong uptake of FDG (arrow) (**a**). ^131^I scintigraphy shows strong uptake in the pelvic mass (arrow) (**b**)

The patient underwent whole-body FDG PET/CT to confirm malignancy and the presence of lymph node or distant metastases. However, there were no lesions with strong FDG uptake (Fig. 2a). Moreover, she underwent abdominal paracentesis several times to confirm malignant cells in the ascitic fluid and reduce the abdominal discomfort due to the massive ascites. However, cytological examinations showed benign mesothelial cells without any malignant cells.

In this case, compared with the typical imaging appearance of epithelial ovarian cancer, the solid component of the pelvic mass had a higher attenuation lesion and calcifications on CT and a relatively smoother margin on MRI. Moreover, the cystic component had various intensities on T2-weighted images, suggesting the possibility of struma ovarii. Therefore, she underwent ^131^I scintigraphy, which showed strong uptake in the normal thyroid and the pelvic mass (Fig. 2b). There were no symptoms or clinical signs of thyroid hormone imbalance. Her free-T3, free-T4, and thyroid stimulating hormone levels were within normal ranges. The preoperative diagnosis was struma ovarii with pseudo-Meigs’ syndrome despite the clinical findings mimicking advanced ovarian cancer.

An exploratory laparotomy was performed for diagnostic and therapeutic purposes, and 3300 ml of yellow serous ascites were evacuated and obtained for cytology. The pelvic mass with dimensions of 10 × 8 × 7 cm^3^ that originated from the left ovary had no capsule rupture and no adhesions. The uterus and right adnexa were unremarkable. There were no enlarged lymph nodes or metastatic and disseminated lesions in the intraperitoneal organs. Left salpingo-oophorectomy was performed for intraoperative diagnosis. On frozen section examination of the left ovarian tumor, struma ovarii was reported. The patient underwent total abdominal hysterectomy and right salpingo-oophorectomy, because the patient and her family members had insisted on it before surgery.

**Fig. 3:**
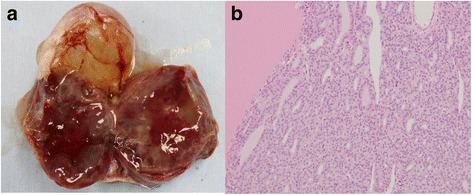
The cut surface of the encapsulated left ovarian tumor shows multiple cysts separated by thickened septa, including the serous and gelatinous yellowish fluid and solid components (**a**). Hematoxylin and eosin-stained paraffin section of the tumor at × 40 magnification shows thyroid follicles filled with various sized colloid lined by cuboidal epithelial cells (**b**)

Gross examination revealed a whitish and partly yellowish encapsulated mass with a slightly irregular surface. The cut surface showed multiple cysts separated by thickened septa, including a serous and gelatinous yellowish fluid and solid components (Fig. 3a). The pathological examination showed that the tumor consisted of thyroid follicles filled with various sized colloid lined by cuboidal epithelial cells (Fig. 3b). There was no evidence of malignancy or other germ cell elements in the specimens. The uterus and right adnexa were histologically unremarkable. Cytological examination of the obtained ascitic fluid showed no malignant cells. The final pathological diagnosis was struma ovarii confined to the left ovary, supporting the preoperative diagnosis.

The patient’s postoperative course was uneventful. The pleural effusion disappeared 8 days after surgery. Serum CA 125 levels decreased to 288 U/ml 4 days after surgery and then returned to normal levels within 2 months. At 6 months, the patient remained disease-free without ascites and pleural effusion.

## Discussion and conclusions

Struma ovarii is a rare specialized form of ovarian teratoma, accounting for only 1% of ovarian neoplasms. More than 50% of the tumor is mature thyroid tissue, although only 5% of patients present with clinical hyperthyroidism [1]. Most cases are asymptomatic and incidentally found during imaging examinations for other purposes. Although this tumor is generally benign, 15–20% of cases present with ascites. This tumor is rarely accompanied by ascites and pleural effusion, which is called pseudo-Meigs’ syndrome. Meigs first described cases of benign ovarian tumors with ascites and hydrothorax in 1937 and proposed limiting true Meigs’ syndrome to benign and solid ovarian tumors including fibroma, thecoma, and granulosa cell tumor [2]. The others, such as benign ovarian cysts, leiomyomas of the uterus, and teratomas including struma ovarii, accompanied by the same symptoms were defined as pseudo-Meigs’ syndrome [3]. Up to the present, some cases of struma ovarii with pseudo-Meigs’ syndrome have been reported and reviewed. However, most cases were preoperatively misdiagnosed as ovarian cancer due to the malignant appearance of the tumor, and unnecessary extended surgery was performed. A precise diagnostic strategy is needed to perform the appropriate treatment.

US, CT, and MRI have been widely used for the differential diagnosis of ovarian tumor. Struma ovarii generally contains solid components or thickened septa in the cystic component, similar to ovarian malignancy. However, it also has some specific imaging features. On US, struma ovarii sometimes shows struma pearl with a smooth, roundish, solid area [4]. On CT, struma ovarii has intracystic high attenuation lesions, indicating the presence of viscid gelatinous colloid material. More than half of the cases show calcifications along the thickened septa or cyst walls. Enhancement of the solid component is variable, depending on the contents of the thyroid tissue [5, 6]. On MRI, the cystic parts show various signal intensities on T2-weighted images depending on the viscosity of the fluid. The solid part is relatively smooth and enhances intensely or moderately [6–8]. In the present case, the tumor had both solid and cystic components separated by thickened septa, like ovarian malignancy. However, the tumor had relatively smooth septa with high attenuation lesions and calcifications on CT. Moreover, the cystic parts showed various signal intensities on T2-weighted images, suggesting that struma ovarii was included in the differential diagnosis. However, mucinous cystic tumor also shows these findings. Thus, accurate diagnosis for struma ovarii by conventional imaging alone remains challenging.

In the review of the literature, the focus was on preoperative diagnosis using imaging modalities and struma ovarii with pseudo-Meigs’ syndrome (Table 1) [1, 9–20] or massive ascites (Table 2) [21–32] and an elevated level of CA125 mimicking advanced ovarian cancer. Most cases were preoperatively misdiagnosed as advanced ovarian cancer based on the findings of conventional imaging. Few cases had a suspected diagnosis of struma ovarii with pseudo-Meigs’ syndrome, although definitive diagnosis was not reached. In most cases, intraoperative diagnosis by frozen section was performed. Many cases with typical struma ovarii were correctly diagnosed intraoperatively. However, some cases were diagnosed incorrectly, leading to unnecessary extended surgery. Moreover, it was more difficult to differentiate between benign and malignant struma ovarii by conventional imaging modalities or intraoperative diagnosis. Thus, the conventional imaging modalities had limitations in the preoperative diagnosis of struma ovarii with pseudo-Meigs’ syndrome, indicating the need for a new diagnostic strategy.

**Table 1:** Literature review of struma ovarii with pseudo-Meigs’ syndrome and elevated CA125 levels

Study	Age (y) Gravidity	Size (cm)	CA125 (U/ml)	Preoperative thyroid function	Imaging modalities	Preoperative diagnosis	Intraoperative diagnosis by frozen section	Treatment
Bethune et al. 1966 [1]	62 multipara	9 × 5 × 5	1621	WNL	X-ray, US, CT	Suspected ovarian malignancy	N/A	ATH + BSO + infracolic OM
Long et al. 2001 [9]	53 multipara	15 × 11 × 7	540	N/A	X-ray, US, CT	Suspected ovarian malignancy	Struma ovarii	ATH + BSO + infracolic OM
	78 multipara	12.2 × 10 × 5.2	124.9	N/A	X-ray, US, CT	Suspected ovarian malignancy	Multilocular cysts lined by columnar epithelium	ATH + BSO
Huh et al. 2002 [10]	65 multipara	5 × 4 × 4	402	Hypothyroidism	US, CT	Suspected ovarian malignancy	Struma ovarii	ATH + BSO + appendectomy + omental biopsy
Loizzi et al. 2005 [11]	65 multipara	7 × 7	161	Hyperthyroidism	X-ray, CT	Suspected ovarian malignancy	Struma ovarii	RSO
Uehara and Sawada 2007 [12]	67 N/A	About 7	2086	WNL	CT	Struma ovarii with Meigs’ syndrome although the ovarian tumor mimicked an advanced malignancy	N/A	ATH + BSO
Obeidat and Amarin 2007 [13]	52 multipara	10 × 15 × 8	149	N/A	X-ray, US	Suspected ovarian malignancy	N/A	ATH + BSO + OM
Mitrou et al. 2008 [14]	55 N/A	22 × 23 × 10	3803	N/A	CT	Suspected ovarian malignancy	N/A	ATH + BSO + infracolic OM + lymph node sampling
Rana et al. 2009 [15]	70 N/A	7.5 × 5.5 × 4	284	WNL	X-ray, US, CT	Suspected ovarian malignancy	N/A	ATH + BSO + partial OM
Jiang et al. 2010 [16]	46 multipara	20 × 18 × 15	1230.9	N/A	CT	Suspected ovarian malignancy	Struma ovarii	ATH + BSO
Obeidat and Saida 2012 [17]	55 multipara	21 × 21 × 9	872	N/A	US, CT, MRI	Suspected ovarian malignancy	N/A	Neoadjuvant chemotherapy (carboplatin and paclitaxel) followed by laparoscopic LSO for obesity
Mostaghel et al. 2012 [18]	72 multipara	9	607.4	N/A	X-ray, US, CT	Suspected ovarian malignancy	Granulosa cell tumor	ATH + BSO + OM
Jin et al. 2015 [19]	52 multipara	7 × 5	1289	N/A	US, CT	Suspected ovarian malignancy	Cystic mature teratoma with a large component of thyroid	ATH + BSO
Present	50 multipara	10 × 8 × 7	1237	WNL	X-ray, US, CT, MRI, FDG PET, ^123^I scintigraphy	Highly suggestive of struma ovarii with pseudo-Meigs’ syndrome	Struma ovarii	ATH + BSO

**Table 2:** Literature review of struma ovarii with massive ascites and elevated CA125 levels

Study	Age (y) Gravidity	Size (cm)	CA125 (U/ml)	Preoperative thyroid function	Imaging modalities	Preoperative diagnosis	Intraoperative diagnosis by frozen section	Treatment
Leung and Hammond 1993 [21]	60 N/A	10	224	N/A	US, CT	Suspected ovarian malignancy	Struma ovarii	ATH + BSO + OM
	77 N/A	8 × 10	2860	N/A	US, laparoscopy		Struma ovarii	ATH + BSO + sampling of enlarged lymph nodes
Jotkowitz and Gee 1993 [22]	79 multipara	N/A	4670	N/A	CT	Suspected ovarian malignancy	Adenocarcinoma	ATH + BSO + OM + multiple peritoneal biopsies
Mancuso et al. 2001 [23]	31 primipara	10 × 9	689	N/A	X-ray, US, MRI	Suspected ovarian malignancy	Struma ovarii	LSO
Loizzi et al. 2002 [24]	83 multipara	10 × 7 × 6.5	1570	N/A	X-ray, CT	Suspected ovarian malignancy	Struma ovarii	ATH + BSO
Bokhari et al. 2003 [25]	51 primipara	15 × 6.5 × 11	1160	N/A	MRI	Suspected ovarian malignancy	Struma ovarii	ATH + BSO + appendectomy
Guida et al. 2005 [26]	42 multipara	12 × 8.5 × 4	2548	Hyperthyroidism	X-ray, US, CT	Suspected ovarian malignancy	Struma ovarii	ATH + BSO + sampling of swollen lymph nodes
Rim et al. 2005 [27]	50 multipara	4 × 4 attached to 3 × 3	878.67	WNL	X-ray, US, CT	Suspected ovarian malignancy	Struma ovarii	ATH + BSO
Paladini et al. 2008 [28]	42 N/A	11 × 7.3 × 8	2548	Hyperthyroidism	US	Suspected ovarian malignancy	Struma ovarii	RSO
Mui et al. 2009 [29]	56 N/A	6 × 5 × 4 coexist with 3	5218	N/A	X-ray, US, CT	Suspected ovarian malignancy	Mature teratoma and struma ovarii	ATH + BSO + OM
Peyron and Coulon 2012 [30]	78 N/A	7	164	N/A	US, MRI	An ovarian malignancy could not be eliminated although suspicious for struma ovarii	Presence of thyroid tissue	Laparoscopic LSO based on intraoperative diagnosis
Sivrioglu et al. 2013 [31]	55 N/A	3	120	WNL	US, CT, MRI	Suspected ovarian malignancy	N/A	N/A
Yadav et al. 2017 [32]	55 multipara	6 × 5 × 4.5	258	N/A	X-ray, US, CT	Suspected ovarian malignancy	Struma ovarii	LSO

For the differential diagnosis of struma ovarii from other ovarian tumors, the usefulness of thyroid scintigraphy or PET using iodine or ^99m^Tc-pertechnetate has been reported. In some cases, the presence of struma ovarii was unexpectedly suspected when thyroid scintigraphy or PET was performed in the follow-up of patients with thyroid cancer [33–37]. In other cases, thyroid scintigraphy was performed for the differential diagnosis of hyperthyroidism, and struma ovarii was incidentally found [38–41]. In only one case, a patient with normal thyroid function underwent thyroid scintigraphy for the differential diagnosis of ovarian tumor based on the findings of conventional imaging [42]. In the present case, struma ovarii was suspected based on the findings of CT and MRI, and the patient underwent ^131^I scintigraphy to confirm the diagnosis. There have been no reports of the diagnosis of struma ovarii with pseudo-Meigs’ syndrome by thyroid scintigraphy. Collectively, these reports suggest that thyroid scintigraphy can be useful to define the diagnosis even in struma ovarii with pseudo-Meigs’ syndrome that mimics advanced ovarian cancer.

However, the differential diagnosis of ovarian tumor with positive thyroid scintigraphy should include malignant struma ovarii and ovarian metastasis from primary thyroid carcinoma. Malignant struma ovarii, defined as thyroid carcinoma arising in struma ovarii, occurs in 5–10% of cases with struma ovarii, and about 30% of cases with malignant struma ovarii have extraovarian spread [43, 44]. In terms of the complications of pseudo-Meigs’ syndrome, there has been only one case report (Table 3) [20]. The usefulness of thyroid scintigraphy for the preoperative diagnosis of malignant struma ovarii is still unclear, because most cases are histologically diagnosed after surgery. The usefulness of thyroid scintigraphy in follow-up or evaluation of radioablation with ^131^I for malignant struma ovarii has been reported [45–48]. However, the sensitivity may be low for detection of residual or recurrent malignant tissue if the thyroid gland is not removed [49]. Ovarian metastasis from primary thyroid carcinoma is extremely rare, and only a few cases have been reported [50–52]. Thyroid scintigraphy shows uptake in the ovarian metastasis from primary thyroid carcinoma, as well as in the malignant struma ovarii. Taken together, although thyroid scintigraphy can be useful for the differential diagnosis of other ovarian tumors, there are a few limitations when attempting to differentiate it from a few rare conditions.

**Table 3:** Literature review of malignant struma ovarii with pseudo-Meigs’ syndrome and elevated CA125 levels

Study	Age (y) Gravidity	Size (cm)	CA125 (U/ml)	Preoperative thyroid function	Imaging modalities	Preoperative diagnosis	Intraoperative diagnosis by frozen section	Treatment
Zannoni et al. 2004 [20]	66 multipara	9.5 × 5.6 × 7	1636	WNL TG: 299.8 ng/ml	US, CT	Suspected ovarian malignancy	Struma ovarii	ATH + BSO + OM + multiple peritoneal biopsies + sampling of pelvic lymph nodes

*WNL* within normal limits, *N/A* Not available, *TG* thyroglobulin, *US* ultrasonography, *CT* computerized tomography, *MRI* magnetic resonance imaging, *FDG* fluorodeoxyglucose, *PET* positron emission tomography, *I* iodine, *ATH* abdominal total hysterectomy, *BSO* bilateral salpingo-oophorectomy, *RSO* right salpingo-oophorectomy, *LSO* left salpingo-oophorectomy, *OM* omentectomy

PET, particularly with ^18^F–FDG as a tracer reflecting cellular metabolism, has been shown to be worth considering alongside conventional imaging modalities. FDG PET has been reported to offer low diagnostic value and have a limited role in differentiating between malignant and benign ovarian tumors due to low ^18^F–FDG uptake, although FDG PET may provide additional diagnostic value for detecting lymph node or distant metastases and suspected recurrences in ovarian cancer [53]. In the differential diagnosis between struma ovarii and malignant struma ovarii or ovarian metastasis from primary thyroid carcinoma, the usefulness of FDG PET has been reported. Benign struma ovarii does not show FDG uptake [34, 37]. However, malignant struma ovarii shows FDG uptake, and this may be useful for evaluation of the biological characteristics and the detection of the metastatic or recurrent lesions [45–47]. There are no reports about the usefulness of FDG PET for ovarian metastasis from primary thyroid carcinoma. However, in the detection of thyroid cancer with suspected recurrence and metastases, FDG PET has been reported to have a sensitivity and specificity of 75–85% and 90%, respectively, while the sensitivity and specificity of thyroid scintigraphy were 53% and 92%. Moreover, the combination of FDG PET and thyroid scintigraphy has been reported to provide better diagnostic value [54]. Collectively, these results suggest that FDG PET may make up for the weakness of thyroid scintigraphy to determine malignant potential in struma ovarii with pseudo-Meigs’ syndrome.

In summary, the combination of thyroid scintigraphy and FDG PET enabled successful preoperative diagnosis of struma ovarii with pseudo-Meigs’ syndrome mimicking advanced ovarian cancer. These modalities can be useful to avoid unnecessary extended surgery and perform non-invasive surgery instead.

## Abbreviations

CT: Computed tomography
FDG: Fluorodeoxyglucose
MRI: Magnetic resonance imaging
PET: Positron emission tomography
Tc: Technetium
US: Ultrasonography
